# The Correlation of the Neutrophil-Lymphocyte Ratio and the Platelet-Lymphocyte Ratio With Pathological Findings in Neuroendocrine Tumors

**DOI:** 10.7759/cureus.17164

**Published:** 2021-08-13

**Authors:** Ozgur Kulahci, Tolga Koseci

**Affiliations:** 1 Department of Pathology, University of Health Sciences, Adana City Education and Research Hospital, Adana, TUR; 2 Department of Medical Oncology, University of Health Sciences, Adana City Education and Research Hospital, Adana, TUR

**Keywords:** neuroendocrine tumours, pathology, neutrophils, lymphocytes, tumor grade

## Abstract

Introduction

The relationship between clinical prognostic factors and blood neutrophil-lymphocyte ratio (NLR) and platelet-lymphocyte ratio (PLR) in some tumors has been investigated. In this study, we examined whether there is a relationship between pathological prognostic factors and NLR as well as PLR only in neuroendocrine tumors (NETs).

Methods

A total of 115 patients with a NET diagnosis between 2014-2020 were included in the study. The efficiency of NLR and PLR in predicting distant metastases was determined by analyzing the receiver operating characteristic (ROC) curve. The relationship between histopathological parameters was also compared.

Results

The cut-off value of NLR was 3.01 for predicting distant metastasis. At this value, the specificity was 73.7%, the sensitivity was 70.7%, and the likelihood ratio was 2.51. There was a significant relationship between NLR and tumor localization, histological grade, mitosis, Ki-67, distant metastasis, and lymphovascular invasion (all p<0.001). The cut-off value of the PLR in predicting distant metastasis was 134.4. At this value, the specificity was 59.6%, the sensitivity was 58.6%, and the likelihood ratio was 1.44. There was no significant relationship between PLR and the histopathological findings (all p>0.05).

Conclusions

In our study, a high histological grade, high mitosis, a high Ki-67 proliferation index, distant metastasis, and lymphovascular invasion were found in patients with NLR at a cut-off value above 3.01. However, we could not attain the same results for PLR. For trucut and endoscopic biopsies in particular, follow-up of patients with grades 1 and 2 NETs along with histopathological findings and evaluation of NLR in peripheral blood may be useful. NLR, which is an easily accessible inflammatory marker, can be used as an independent predictive factor in NETs.

## Introduction

Neuroendocrine tumors (NETs) originate from neuroendocrine cells with characteristic histopathological and immunohistochemical properties [1]. NETs can appear in different parts of the body, including the gastrointestinal system, respiratory system, central nervous system, larynx, thyroid, breasts, skin, and urogenital system [2]. They most commonly occur in the gastrointestinal tract, lungs, and pancreas. It is thought that a systemic inflammatory response is associated with poor prognosis in many cancers in terms of predicting tumor invasion, metastasis, and angiogenesis [3]. Recently, the blood neutrophil-lymphocyte ratio (NLR) and the platelet-lymphocyte ratio (PLR) have been investigated in relation to poor prognosis in various oncological tumors [4,5]. Because they can be easily measured in cancer patients, NLR and PLR in peripheral blood have been examined in predicting prognostic results and risk classification before treatment. A high NLR has been associated with advanced stage and poor prognosis in many tumor types, such as stomach and breast cancers [6,7]. In patients with thrombocytosis, a connection has been established between poor prognosis and shorter survival in various tumor types, such as lung, colon, gastric, breast, and ovarian cancers [8]. Various studies have been conducted involving NLR and PLR regarding prognosis in NETs. A relationship was determined between a high NLR and large tumor size, an advanced stage, a high grade, as well as decreased survival time in NETs [9]. In another study, the preoperative NLR was determined as a potential independent marker for lymph node metastasis and recurrence-free survival [10]. While there are studies indicating an increase in NLR and PLR as the grade increases in NETs, some other studies have indicated no relationship between the increase in PLR and metastasis [11,12]. Studies showing the relationship between NLR, PLR, and NETs are generally related to clinical parameters and survival. This study was planned based on histopathological parameters. The aim was to determine the relationship between NLR and PLR in the peripheral blood and pathological prognostic factors in NETs. The cut-off values were determined for predicting distant metastasis by analyzing the receiver operating characteristic (ROC) curve for NLR and PLR. Its relationship with histopathological parameters was also evaluated.

## Materials and methods

Pathological evaluation

A total of 115 patients with NETS who were seen between 2014-2020 in our hospital’s pathology department were retrospectively analyzed. Approval for the study was obtained from the University of Health Sciences, Adana City Education and Research Hospital Ethics Committee (Decision no: 957). Patients who were diagnosed with NET via tru-cut biopsy, endoscopic biopsy, colonoscopic biopsy, excisional biopsy, or resection materials were re-evaluated by an experienced pathologist. For cases of NETs with metastases on imaging, those with a diagnosis of metastasis that was confirmed by biopsy or tumor resection were included in the study. Patients who were not diagnosed with a biopsy, those who had a recurrent tumor and received chemoradiotherapy were excluded from the study. Age, gender, tumor localization, histological grade, mitosis, distant metastasis, lymphovascular invasion, perineural invasion, immunohistochemical Ki-67, synaptophysin and chromogranin A staining, and a correlation between NLR and PLR in peripheral blood were evaluated in all patients. Tumor localizations were grouped into those in the gastroenteropancreatic system (esophagus, stomach, small intestine, large intestine, appendix, pancreas), lungs, and other organs.

The histological grade of NET was evaluated according to the 2019 World Health Organization (WHO) classification of digestive system tumors and the 2015 WHO classification of lungs, pleura, thymus, and heart tumors. For histological grading of the gastroenteropancreatic system and other localizations, it was grouped either as a grade 1 well-differentiated NET, a grade 2 well-differentiated NET, or a grade 3 well-differentiated NET [13]. Typical carcinoid tumors were considered as grade 1 in the lung; atypical carcinoid tumors were considered as grade 2, and neuroendocrine carcinomas were considered as grade 3 [14]. According to the WHO classification, mitosis was grouped as less than 2 in 2 mm^2^, between 2-20, and more than 20 upon microscopic examination for the gastroenteropancreatic system and other localizations [13]. For the lung, it was grouped as less than 2 in 2 mm^2^, between 2-10, and more than 10 [14]. If the biopsy showed less than 2 mm^2^, the entire tissue was evaluated. Immunohistochemical stains were completed with Bond-Max fully automated IHC and ISH (Leica Biosystems Division of Leica Microsystems Inc, Buffalo Grove, IL) according to the manufacturer’s staining procedure. Ki-67 (Novocastra™ Liquid Mouse Monoclonal Antibody Ki-67 Antigen, Leica Biosystems Newcastle Ltd, Newcastle Upon Tyne, UK), which was used for the gastroenteropancreatic system and other localizations, was grouped as less than 3% upon microscopic examination, between 3-20%, and greater than 20%. It was grouped at up to 5%, between 5-20%, and greater than 20% for the lung. Ki-67-stained slides were counted manually with images taken with the camera and then printed out in color. Each tumor slide was manually scanned with a microscope at x10 objective. The largest Ki-67 positivity (hot spot) area was chosen for photography and printing. The color image of the hot spot was captured with the camera snapshot. It was printed on white paper. Ki-67-positive dark brown tumor nuclei were counted and the Ki-67 proliferation index was calculated. Light brown or pale staining nuclei were ignored during counting. Distant metastasis, lymphovascular invasion, perineural invasion, synaptophysin (NCL-L-SYNAP-299, Leica Biosystems Newcastle Ltd), and chromogranin A (NCL-L-CHROM-430, Leica Biosystems Newcastle Ltd) were grouped as negative and positive. The blood values of patients taken from the peripheral vein within two weeks before biopsy or surgery were analyzed with a Beckman Coulter DXH-800 (Beckman Coulter Eurocenter, Nyon, Switzerland). Neutrophil, platelet, and lymphocyte counts were determined. NLR was calculated by dividing the neutrophil count by the lymphocyte count. PLR was calculated by dividing the platelet count by the lymphocyte count. An ROC curve analysis was performed with NLR and PLR to determine the relationship between cut-off values, and the above pathological parameters for predicting metastasis were compared.

Statistical analysis

SPSS Statistics version 19 was used for statistical evaluation of the data obtained in the study (IBM, Armonk, NY). Descriptive statistics related to variables such as age, NLR, and PLR were presented as the median (range). Categorical variables such as gender, tumor localization, mitosis, Ki-67 index, histological grade, distant metastasis, lymphovascular invasion, perineural invasion, and synaptophysin and chromogranin A staining were presented in numbers (n) and percentages (%). An ROC curve analysis was performed to determine the cut-off value in predicting metastasis of NLR and PLR. The area values under the curve that were obtained from the ROC curve analysis were used to compare predictive activities of NLR and PLR. Categorical variables were analyzed using the Chi-squared test, Fisher's exact test, or likelihood ratio test. Sensitivity, specificity, and likelihood ratio parameters were used to investigate the accuracy of diagnostic tests for distant metastasis with NLR and PLR. A p-value <0.05 was considered statistically significant.

## Results

All included patients were between the ages of 11 and 88 years; two cases were under the age of 18, and their tumors were located in the appendix. While 47.8% of the patients were female, 52.2% were male, and 56.5% of the tumors were localized in the gastroenteropancreatic system. Distant metastases were present in 49.6% of the patients. Categorical variables were analyzed using the Chi-squared test, Fisher's exact test, or likelihood ratio test. The clinical and pathological features of NET patients are shown in Table 1.

**Table 1:** Clinical and pathological features of patients with NET *Values in parentheses for mitosis and Ki-67 indicate the lungs NET: neuroendocrine tumor; GEP: gastroenteropancreatic; LVI: lymphovascular invasion; PNI: perineural invasion; NLR: neutrophil-lymphocyte ratio; PLR: platelet-lymphocyte ratio

Variable	All patients, n (%)
Age in years, median (range)	61 (11-88)
Male gender	60 (52.2)
Localization	
GEP system	65 (56.5)
Lung	35 (30.4)
Other	15 (13)
Histological grade (G)	
G1	44 (38.3)
G2	12 (10.4)
G3	59 (51.3)
Mitosis*	
<2	44 (38.3)
2-20 (2-10)	12 (10.4)
>20 (>10)	59 (51.3)
Ki-67*	
<3 (5)%	44 (38.3)
3-20 (5-20)%	12 (10.4)
>20%	59 (51.3)
Metastasis	
Negative	58 (50.4)
Positive	57 (49.6)
LVI	
Negative	47 (40.9)
Positive	68 (59.1)
PNI	
Negative	92 (80)
Positive	23 (20)
Synaptophysin	
Negative	2 (1.7)
Positive	113 (98.3)
Chromogranin A	
Negative	12 (10.4)
Positive	103 (89.6)
NLR, median (range)	3.04 (0.73-52.61)
PLR, median (range)	135 (14-828)

An ROC curve analysis was performed for NLR and PLR. The area under the curve in NLR was 75.6%, and the confidence interval was 66.7-84.6%. The cut-off value of NLR was 3.01 in predicting distant metastasis. At this value, the specificity was 73.7%, the sensitivity was 70.7%, and the likelihood ratio was 2.51. The area under the curve in the PLR was 63.1%, and the confidence interval was 52.4-73.7%. The cut-off value of the PLR for predicting distant metastasis was 134.4. At this value, the specificity was 59.6%, the sensitivity was 58.6%, and the likelihood ratio was 1.44.

A correlation was found between the cut-off value of NLR and tumor localization, histological grade, mitosis, Ki-67 proliferation index, metastasis, and lymphovascular invasion (all p<0.001). A higher histological grade, high mitosis, and a high Ki-67 proliferation index were determined in the group with an NLR cut-off value above 3.01. This group was mostly located in the lungs and other tissues, and it was more in distant metastasis and lymphovascular invasion. There was no correlation between NLR cut-off value and perineural invasion and synaptophysin and chromogranin A immunohistochemical staining (all p>0.05). The correlation between NLR and categorical variables is shown in Table 2.

**Table 2:** Correlation between NLR in peripheral blood and histopathological findings *Values in parentheses for mitosis and Ki-67 indicate the lungs GEP: gastroenteropancreatic; LVI: lymphovascular invasion; PNI: perineural invasion; NLR: neutrophil-lymphocyte ratio

	NLR <3.01, n (%)	NLR >3.01, n (%)	P-value
Localization			<0.001
GEP system	42 (64.6)	23 (35.4)	
Lung	13 (37.1)	22 (62.9)	
Other	2 (13.3)	13 (86.7)	
Histological grade (G)			<0.001
G1	36 (81.8)	8 (18.2)	
G2	3 (25)	9 (75)	
G3	18 (30.5)	41 (69.5)	
Mitosis*			<0.001
<2	36 (81.8)	8 (18.2)	
2-20 (2-10)	3 (25)	9 (75)	
>20 (>10)	18 (30.5)	41 (69.5)	
Ki-67*			<0.001
<3 (5)%	36 (81.8)	8 (18.2)	
3-20 (5-20)%	3 (25)	9 (75)	
>20%	18 (30.5)	41 (69.5)	
Metastasis			<0.001
Negative	42 (72.4)	16 (27.6)	
Positive	15 (26.3)	42 (73.7)	
LVI			<0.001
Negative	37 (78.7)	10 (21.3)	
Positive	20 (29.4)	48 (70.6)	
PNI			0.113
Negative	49 (53.3)	43 (46.7)	
Positive	8 (34.8)	15 (65.2)	
Synaptophysin			0,99
Negative	1 (50)	1 (50)	
Positive	56 (49.6)	57 (50.4)	
Chromogranin A			0.235
Negative	4 (33.3)	8 (66.7)	
Positive	53 (51.5)	50 (48.5)	

No relation was determined between the cut-off value of PLR and tumor localization, histological grade, mitosis, Ki-67 proliferation index, metastasis, lymphovascular invasion, perineural invasion, synaptophysin, and chromogranin A immunohistochemical staining (all p>0.05). The correlation between PLR and the categorical variables is summarized in Table 3.

**Table 3:** Correlation between PLR in peripheral blood and histopathological findings *Values in parentheses for mitosis and Ki-67 indicate the lungs GEP: gastroenteropancreatic; LVI: lymphovascular invasion; PNI: perineural invasion; PLR: platelet-lymphocyte ratio

	PLR <134.4, n (%)	PLR >134.4, n (%)	P-value
Localization			0.217
GEP system	36 (55.4)	29 (44.6)	
Lung	15 (42.9)	20 (57.1)	
Other	5 (33.3)	10 (66.7)	
Histological grade (G)			0.086
G1	27 (61.4)	17 (38.6)	
G2	4 (33.3)	8 (66.7)	
G3	25 (42.4)	34 (57.6)	
Mitosis*			0.086
<2	27 (61.4)	17 (38.6)	
2-20 (2-10)	4 (33.3)	8 (66.7)	
>20 (>10)	25 (42.4)	34 (57.6)	
Ki-67*			0.086
<3 (5)%	27 (61.4)	17 (38.6)	
3-20 (5-20)%	4 (33.3)	8 (66.7)	
>20%	25 (42.4)	34 (57.6)	
Metastasis			0.076
Negative	33 (59.6)	25 (43.1)	
Positive	23 (40.4)	34 (59.6)	
LVI			0.119
Negative	27 (57.4)	20 (42.6)	
Positive	29 (42.6)	39 (57.4)	
PNI			0.926
Negative	45 (48.9)	47 (51.1)	
Positive	11 (47.8)	12 (52.2)	
Synaptophysin			0.165
Negative	0	2 (100)	
Positive	56 (49.6)	57 (50.4)	
Chromogranin A			0.607
Negative	5 (41.7)	7 (58.3)	
Positive	51 (49.5)	52 (50.5)	

**Figure 1:**
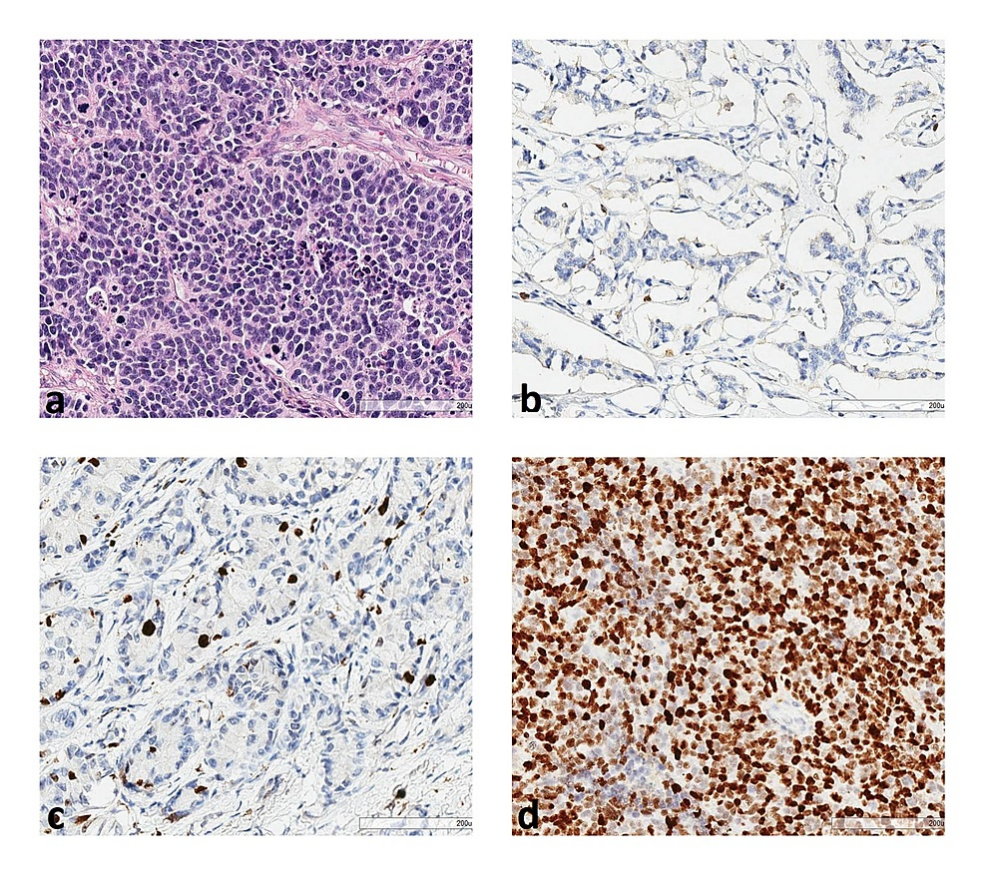
(a) Grade 3 neuroendocrine carcinoma (hematoxylin and eosin 200x); (b) Grade 1 NET (Ki-67 200x); (c) Grade 2 NET (Ki-67 200x); and (d) Grade 3 NET (Ki-67 200x) immunohistochemical Ki-67 staining NET: neuroendocrine tumor

Microscopic images of grade 3 NET hematoxylin-eosin staining and Ki-67 immunohistochemical staining of grade 1, 2, and 3 NETs are shown in Figure 1.

## Discussion

Newly diagnosed NETs account for ~0.5% of all malignancies. In a study conducted in the Netherlands, this type of tumor was mostly seen in the gastrointestinal system (62-67%) and lungs (22-27%) [15]. The estimated prevalence of gastroenteropancreatic NETs is 35/100,000 people [16]. NETs comprise 2-5% of all pancreatic tumors and 25% of primary lung neoplasms [17]. In addition, 20% of NETs in the lung are small cell lung cancer, 3% are large cell neuroendocrine carcinoma, 1.8% are typical carcinoid tumors, and 0.2% are atypical carcinoid tumors [17,18]. Gastroenteropancreatic and lung NETs are graded according to histopathological features, mitosis rate, and the Ki-67 proliferation index [13,14]. NET cells have a uniform, round-oval small nuclei, inconspicuous nucleoli, and a chromatin pattern defined as salt and pepper in appearance [14]. NETs are tumors that immunohistochemically express neuroendocrine markers, such as chromogranin A and synaptophysin, and they characteristically show organoid, nesting, trabecular, or gyriform/serpentine growth patterns [19].

There are studies indicating that 12-22% of NETs have metastases during the first diagnosis [15]. In our study, there was a metastasis rate of 49.6%, which presented mostly as liver, brain, and bone metastasis and was due to the higher proportion of NETs in the lungs and other organs and higher histological grades. Distant metastasis and local recurrence are the leading causes of death in patients with malignant neoplasia [20]. Inflammation contributes to the proliferation of malignant cells, angiogenesis, and metastasis in patients with tumors [21]. Inflammatory cells create a favorable environment for tumor growth early in the neoplastic process, facilitate genomic instability, and support angiogenesis. They then stimulate inflammatory mechanisms in tumor formation, such as selection-ligand interactions, matrix metalloprotein production, and chemokine functions, to support the neoplastic spread and metastasis [22]. Neutrophils can secrete various cytokines, such as granulocyte-macrophage colony-stimulating factor (GM-CSF), tumor necrosis factor-alpha (TNF-alpha), interleukin-1 (IL-1), and IL-8, to increase tumor formation, spread, and metastasis [23]. In addition, neutrophils can facilitate angiogenesis and metastasis by increasing tumor cell adhesion to the endothelium [24]. Neoplastic cells contain various membrane receptors that can bind directly to and activate platelets. Notably, platelets have been found to include factors that contribute to tumor growth, angiogenesis, and metastasis [25]. It has also been reported that systemic inflammation is associated with poor survival in various cancers [26].

A literature search yielded few studies comparing NLR and PLR with only histopathological parameters in NETs throughout the body. In our study, while there was a relationship between NETs in the lungs and other organs and NLR in terms of localization, we could not determine such relationships with PLR. However, because we have been unable to identify similar studies in the literature, further research on this subject may be beneficial. In various studies, the relationship between NLR and histological grade, lymphovascular invasion, and the Ki-67 proliferation index was determined in NETs. In these studies, the cut-off value for NLR was 2.4, 2.056, 2.2, and 2.31, respectively [9,10,20,27]. In another study, the NLR cut-off value was 2.8, but no relation was determined with the grade [28]. In our study, a high histological grade, a high Ki-67 proliferation index, high mitosis, and more lymphovascular invasion were determined in patients with cut-off values over 3.01 with NETs. Some studies, similar to ours, have found no relationship between NLR and perineural invasion [9]. In NETs, the relationship between PLR and grade was found in some studies with the cut-off values of 151.4 and 142, respectively [27,28]. In our study, no relation was found between PLR and histological grade, mitosis, the Ki-67 proliferation index, lymphovascular invasion, and perineural invasion. In pancreatic NETs, a relationship was found between patients whose NLR cut-off value was 2.4 and their TNM stage [9]. In NETs, the relationship between NLR and metastasis was determined in studies with a cut-off value of 2.2 and 2.8 [20,28]. In our study, distant metastases were detected in patients with an NLR cut-off value above 3.01. As in another study with a PLR cut-off value of 142, in our study, no relation was found between distant metastasis when the cut-off value was 134.4 [28]. The diagnosis of NETs can be made by biopsy from the primary organ or metastasis. Therefore, it is critical to investigate the factors related to metastases. Preoperative factors due to distant metastases in NETs are not well defined. Systemic inflammation markers such as NLR and PLR, which are easy to calculate, are advantageous in this respect.

In studies with NETs in the gastric system, pancreas, and lungs, the researchers mostly compared only clinical parameters and survival in these organs with NLR and/or PLR. Our study, which explores NETs in all systems and is based on pathological parameters, differs from those studies in this respect. Survival, lymph node metastasis, and distant metastases actually depend on the histopathological features of the tumor. Cut-off values are important for NLR and PLR. In some studies, cut-off values for NLR and PLR were determined empirically. In many studies, 5.0 was used as the cut-off value for NLR, while the cut-off value for PLR ranged from 150 to 300 [29]. In our study, the cut-off value was calculated with an ROC curve based on predicting distant metastasis for NLR and PLR. The best cut-off values for NLR and PLR were 3.01 and 134.4, respectively. The area under the curve was 75.6% and 63.1%, respectively. These values show that NLR alone is superior to PLR as an independent predictive factor.

Our study had some limitations, including the use of a single center, the small number of cases, and the higher grade of tumors in the lungs and other organs. However, our review of NETs throughout the body and the use of histopathological parameters differentiate it from other studies.

## Conclusions

In NETs, the diagnosis is commonly made by tru-cut, endoscopic, and bronchoscopic biopsies. The tumor area to be evaluated microscopically may be less than 2 mm^2^ in biopsies. Therefore, in small biopsies, the microscopic area where Ki-67 and mitosis will be evaluated, which is important in determining the histological degree, may be insufficient. This is especially important in the differential diagnosis of grade 1 and grade 2 well-differentiated NETs. The evaluation of NLR in peripheral blood together with histopathological findings may be useful in follow-ups, especially for patients with a grade 1 or grade 2 NET diagnosis. In our study, a high histological grade, high mitosis, a high Ki-67 proliferation index, distant metastasis, and lymphovascular invasion were found in patients with an NLR cut-off value above 3.01. NLR, which is an easily accessible inflammatory marker, can be used as an independent predictive factor in NETs. However, we could not achieve similar results for PLR. NLR above certain values can be used for high-risk NET patients who need special treatment and close follow-up.
